# Spatiotemporal variation in travel regularity through transit user profiling

**DOI:** 10.1007/s11116-016-9747-x

**Published:** 2016-11-10

**Authors:** Ed Manley, Chen Zhong, Michael Batty

**Affiliations:** Centre for Advanced Spatial Analysis, University College London, Gower Street, London, WC1E 6BT UK

**Keywords:** Public transportation, Regularity, Smart card data, Clustering, Transport dynamics

## Abstract

New smart card datasets are providing new opportunities to explore travel behaviour in much greater depth than anything accomplished hitherto. Part of this quest involves measuring the great array of regular patterns within such data and explaining these relative to less regular patterns which have often been treated in the past as noise. Here we use a simple method called DBSCAN to identify clusters of travel events associated with particular individuals whose behaviour over space and time is captured by smart card data. Our dataset is a sequence of three months of data recording when and where individual travellers start and end rail and bus travel in Greater London. This dataset contains some 640 million transactions during the period of analysis we have chosen and it enables us to begin a search for regularities at the most basic level. We first define measures of regularity in terms of the proportions of events associated with temporal, modal (rail and bus), and service regularity clusters, revealing that the frequency distributions of these clusters follow skewed distributions with different means and variances. The analysis then continues to examine how regularity relative to irregular travel across space, demonstrating high regularities in the origins of trips in the suburbs contrasted with high regularities in the destinations in central London. This analysis sets the agenda for future research into how we capture and measure the differences between regular and irregular travel which we discuss by way of conclusion.

## Introduction

At coarse spatial and temporal scales, urban transportation systems might be assumed to be an amalgam of highly routinised traveller behaviours. During each day, barring major events or disruptions, travel demand rises and falls in broadly predictable ways, predominantly aligning with patterns of work-related travel at the beginning and end of the working day and from suburbs to city centres and core workplaces and back. Yet substantial explorations of travel behaviour have shown that the nature of such regularity is considerably more complex, varying within and between individuals on a day-to-day basis (Hanson and Huff 1986), influenced by socio-economic status and land use activity types (Zhong et al. 2015), trip chaining (Primerano et al. 2008), changes in weather (Saneinejad et al. 2012), and simple ad hoc requirements such as managing congestion through physical means. Rather than assuming the widespread persistence of routine behaviours, transportation system dynamics can be better understood by capturing how regularity of persists at the individual scale. Through various measures of individual regularity with respect to the occurrence of trip-making, it is possible to explore how these dynamics can vary widely over space and time.

Regularity in travel behaviour has been an active research area ever since the first surveys of household transportation usage were carried out over half a century ago. In the 1970s and 1980s, diurnal activities which generated trips in households within the framework of time budgeting led to many diary-based surveys which demonstrated the existence of day-to-day variability in travel activity, and thus highlighted limitations in trip generation models built using peak hour or entire day survey approaches (Hanson and Huff 1986; Huff and Hanson 1986; Hanson and Huff 1988; Pas 1987; Pas and Koppelman 1986; Jones and Clarke 1988). Variability in departure times (Mahmassani and Chang 1985), route choice (Mahmassani and Stephan 1988), mode choice (Mannering et al. 1994), the regularity activity engagement (Minnen et al. 2015), and temporal trip rates (Pas and Sundar 1995) were investigated. More recently, mobility data through GPS traces has enabled the automation and extension of longitudinal travel behaviour analysis (Pendyala 1999; Axhausen et al. 2002). Through these data, measures of social travel (Schlich and Axhausen 2003; Schlich et al. 2004), the extraction of activities and land use related to trip-making (Huang et al. 2010; Zhong et al. 2015) and the definition of individual activity spaces (Schönfelder and Axhausen 2003; Schönfelder et al. 2006) have been explored.

While conventional approaches yield useful insights into the nature of regular travel behaviour, the typical scale of data collection means they inevitably fail to capture wider variation in behaviours induced by the social, spatial and environmental context (Van Acker et al. 2010). At present, there is little understanding of large-scale spatiotemporal variation in regularity behaviour. Newly available, large-scale mobility datasets do, however, provide significant opportunities for widening insights into travel behaviour. These datasets capture the mobility of large proportions of the travelling population at fine spatial and temporal scales, usually focussing on the homogeneity and heterogeneity of traveller behaviours at many temporal scales but all constructed from the very fine scale data that is streamed in real time captured from automated devices. In recent years, travel behaviour research has been strengthened through analysis of large volumes of smart card transaction data (Morency et al. 2006; Pelletier et al. 2011; Long and Thill 2015; Zhong et al. 2016), GPS route traces (Manley et al. 2015; Wu et al. 2014), and mobile phone movement data (Gonzalez et al. 2008; Schneider et al. 2013; Ahas et al. 2015). Yet less well researched are the opportunities these data afford in analysing longitudinal behaviours. Through collection of mobility trends at the level of the individual traveller, new potential for profiling travellers and observing changes in behaviour over time have emerged. The analysis of traveller profiles from such data is the main goal of this paper.

Although data volumes in transportation analysis tend to be large due to the fact that the size of potential movements between many locations has always been substantial long before the age of real-time streamed data, it has not been possible to capture the kind of diversity in trip-making that is associated with many spatial and temporal scales until quite recently. Transportation data such as that generated by individuals activating sensors which capture their locations and movements enables behaviour at all scales to be examined and only now is it possible to define and visualise the enormous heterogeneity in traveller behaviour from such datasets. In short, this kind of ‘big’ data forces us to recognise that regularities and irregularities which are related to extreme events as well as variations in individual behaviour must be separated out from more general noise in such data if we are to produce a better understanding of the reasons why and when people travel.

Regularity is thus only interesting if we are able to define this as a series of baselines against which to compare more ad hoc travel such as that pertaining to disruptions, infrequent and extreme events, individual variations in generic travel behaviour and variations across different modes, temporal and spatial locations. Such an understanding is essential for policy making which must build on the deepest understanding of travel dynamics that we can determine. We also need to determine the regularities associated with individual travellers and their aggregation into groups of like-behaviours. In short we need to explain how individual regularities add up to collective regularities and the way such regularities become irregular as we aggregate and disaggregate over space and time. We need to achieve this kind of understanding so we can target policies for improving the operation of the system and the travel experience for passengers at the right spatial and temporal scales. As we will see, the data from the smart cards used for fare collection on public transport in London (called the Oyster card) is able to provide us individual-level long-term views of traveller behaviours. This provides a potential view on travel regularity at spatial and temporal scales that have not been previously possible through conventional datasets. These insights can yield benefits for transport planners, where differentiation between regular and ad hoc travel can contribute towards shaping how services are planned and operations amended, responsive to different types of travel behaviour.

As we have individual data on where a person begins and ends a trip, we can search for regularities across any spatial and temporal scale with respect to the volume of travellers at different points in space and time–stations and times of entry and exit. Such as in (Gong et al. 2012), peak hours and differences between weekday and weekend are analysed using smartcard data from Shenzhen. Zhong et al. (2015b) further extend such analysis from three levels-trips, stations and urban system, demonstrated using data from Singapore. More examples can be found from (Pelletier et al. 2011; Yue et al. 2014). There are literally thousands of possible kinds of regularity to be explained when we have individual data at the most micro level, but in this paper, we will restrict ourselves to regularities that pertain to particular windows of time and to locations, not to aggregations of different windows or to sets of locations which imply actual trips between origins and destinations. In fact, we will restrict our analysis in this paper to regularities associated with entries and exits from the transit systems but not regularities associated with actual trips where we need to match exactly entries with exits for individual travellers, nor regularities that pertain to trips by a single individual during the day such as commuting to and from work.

To an extent, our analysis is a search for unique clusters in a time series and as such it is subject to the usual problems of ecological fallacy or modifiable aggregation which imply different kinds of regularity at different scales. But in this case, we are not searching for invariance in regularities and in any case our analysis is guided by what we consider to be plausible with respect to daily and weekly, possibly monthly travel behaviour. We envisage in fact that we will uncover different processes at different scales. In short our analysis is of regular volumes of travellers at points or windows in time or in space but not volumes that pertain to movements through the system *per se*. Our focus is thus on location rather than spatial interaction and in this sense, this paper is a prelude to a more exhaustive analysis which deals with regularities in movement. In fact, it is possible to imply some movement from regularities in event occurrence at different points in time, such as the usual peak hour travel patterns, but these are not formally calculated in this paper.

To determine regularities, we use a very straightforward technique of clustering individual behaviours in terms of when they take place over days and in particular locations using criteria that is plausible with respect to evaluating the degree of similarity between events. The method we use is called DBSCAN which was first developed by Ester et al. (1996) and is a simple method of relating events—where in this case a traveller registers at an origin entry or destination exit—which are close to one another in terms of their temporal distance from one another. The method also assumes that there is a minimum size to the cluster and in this way, a simple algorithm is able to extract unique clusters from any dataset whose events are ordered in linear time—such as the sequence of tap-in or tap-outs on a transit system. The DBSCAN method has been used for clustering travel data in Brisbane (Kieu et al. 2014) and in Beijing (Ma et al. 2013) which provide good comparisons with the analysis of profiles here. This paper extends these earlier applications by measuring how regularity unfolds across space and time. For capturing clusters in our transit data, we will detail how the method works in detail when we discuss the analysis below.

Thus the purpose of our preliminary analysis is to analyse large-scale variation in regular and irregular travel behaviour. Building upwards on individual-level smart card travel transaction data, it is possible to derive a system-wide spatiotemporal understanding of regularity in travel behaviours. In the first instance, the paper will describe the dataset and context within which the study is conducted. Following this, “capturing traveller regularity” section provides a description of the computational method used to capture individual-level regularity, outlining three different definitions of travel regularity. Using these definitions, the paper undertakes two stages of analysis. “Regularity at different scales” section addresses the nature of regularity according to each definition, examining its persistence and nature across all travellers over space and time. “Spatiotemporal variation in regularity and irregularity” section goes deeper into the existence of regularity relative to non-regular behaviours, providing insight into the causes of regularity, showing the influences of demographics, land use, work place patterns, and major events. The paper will conclude with a discussion of how this view of travel behaviour can aid in planning and modelling the transportation system, and how these methods might be refined and extended in future research.

## Context and dataset

This study is based on an archive of public transportation smart card transactions data recorded for public transport in Greater London (UK) between June 16th 2012 and September 9th 2012. Smart cards—known in London as Oyster cards—allow travellers to enter and exit the transportation system having paid their fare prior to use of the system, these monies being recorded on the smart card. The Oyster card system is used by around 85% of all travellers on the London Underground and bus network although this percentage varies during the day as it is still possible to purchase traditional paper tickets for use on National Rail and London Transport outside the London region (Transport for London 2013).

As travellers enter and exit the system, transactions are recorded that provide details about the type of trip that has been undertaken. Each transaction record includes references to type of card, travel mode, date and time of travel, trip origin or destination (in the cases of rail and underground travel), and the service choice (where trains or buses are taken). These data are recorded for each traveller, denoted by a unique, but anonymised, identifier. The Oyster card is little different to the other smart card data sets that introduced in (Agard et al. 2006), and more details can be refer to (Gordon et al. 2013).

Given the personal nature of the data, no additional metadata is provided in relation to the traveller holding the card. This limits the potential depth of analysis, for instance, travel behaviour relative to home and work locations has to be derived through inferring methods (Zhong et al. 2014); demographic attributes to a user has been enriched through integration of the other data sets (Long and Thill 2015), however, its reliability needs further investigated given the relative sparsity of the network. Unlike these approaches, this paper deepens the analysis focusing on making use of the best features of the data itself (long-term, large volume and good coverage) through a systematic framework.

The study uses transaction data recorded across 49 weekdays over the approximate 3-month period including the 2 weeks of the Olympics Games in London which has been excluded from this study. The dataset contains 639,979,438 transaction records, averaging at around 13 million transactions per day, for 11,535,090 individual travellers. Transactions are classified into ‘tap-in’ events at the start of rail journeys, and located at a specific rail station, ‘tap-out’ events, which mark the end of a journey (and can be linked to ‘tap-in’ events), and bus journey events, which are provided with the bus service identity but are not spatially located. Table 1 provides a breakdown of the occurrence of each frequency type.

**Table 1:** Frequencies of transaction types used within the study

Transaction type	Count	Proportion
All transactions	639,979,438	1.0
All tap-in transactions	181,662,481	0.28
All tap-out transactions	180,132,354	0.28
All bus transactions	276,474,597	0.43

Note that in the rest of this paper we will refer to volumes which number in the millions in shorthand form as 639.979 m or round the data up or down to integer millions

## Capturing traveller regularity

The purpose of our research is to identify and analyse the regular behaviour of each distinct traveller. As we noted above, regularity in travel behaviour can take many forms, dependent on simply travel time, travel mode, or a specific location, so it is important that the methods used in identifying regularity remain flexible to these variations. The method should impose as few assumptions on how regularity is manifested as possible. In order to avoid placing specific constraints on the nature of regularity, the simple temporal clustering approach noted above is adopted. Clustering allows us to identify high density events in temporal activity, indicative of regular behaviour. Various approaches can be adopted, but the method most closely aligned with the requirements stated above is DBSCAN (Density-based spatial clustering of applications with noise) (Ester et al. 1996). DBSCAN places few constraints on how a cluster must appear by building clusters based solely on the spatial density of data points, in contrast to other clustering methods that require a predefined specification of cluster size (e.g. k-means clustering) or assume hierarchical linkage between clusters.

The DBSCAN method requires the specification of two parameters, both of which are related to the required density of data points that constitute a cluster. The first is a definition of the minimum number of points (*minPt*) that a point must be *near to* in order for the point to be deemed to be within a cluster, and the second is a specification of the temporal distance (*dist*) threshold within which two points would be classified as *near* to each other. Although different measures have been tested, for brevity this paper will not explore the impact of variations in these parameters on cluster sizes and definitions of regularity, we consider these values that we have chosen to be optimal. Through these tests, a combination of *dist* = 30 min and *minPt* = 5 produced clusters that aligned well with observations within the data of regularity and repeated journeys, producing intuitively acceptable and plausible clusters of events. While these parameter settings may be applicable elsewhere, it should be noted that these were set specifically for the range and size of the study dataset based on traveller behaviour in Greater London. As we show elsewhere in a comparison of Beijing, London and Singapore (Zhong et al. 2016), there are differences in diurnal variability of travel volumes in these different cities and it is likely that these would be reflected in the parameters which produce optimal regularities. We have not tested for this variability on these different datasets as yet.

The specification of the clustering algorithm is only one element, however. Consideration must also be given to the type of travel behaviour being clustered. Within the Oyster card data, a differentiation is made between trip starts (or ‘tap-ins’) on the Underground and rail system, trip ends (or ‘tap-outs’) on the Underground or rail, and bus journeys which only register the event of being on a bus. Each record has a time associated with it, and a station or bus service at which the travel record was generated. In order to again retain flexibility in our analysis of regularity, it was decided that clustering would be applied in three ways—firstly, to all transactions, regardless of type; second, by transaction type, regardless of location; and third, by specific transaction at a specific location (station or service). This differentiation provides variation in the specificity of the regularity definition, maintaining flexibility in the definition, but furthermore enables insight into how far regularity persists despite increasing specificity. As we implied earlier, we consider these to be the most fruitful elements of regularity in this preliminary study.

**Fig. 1:**
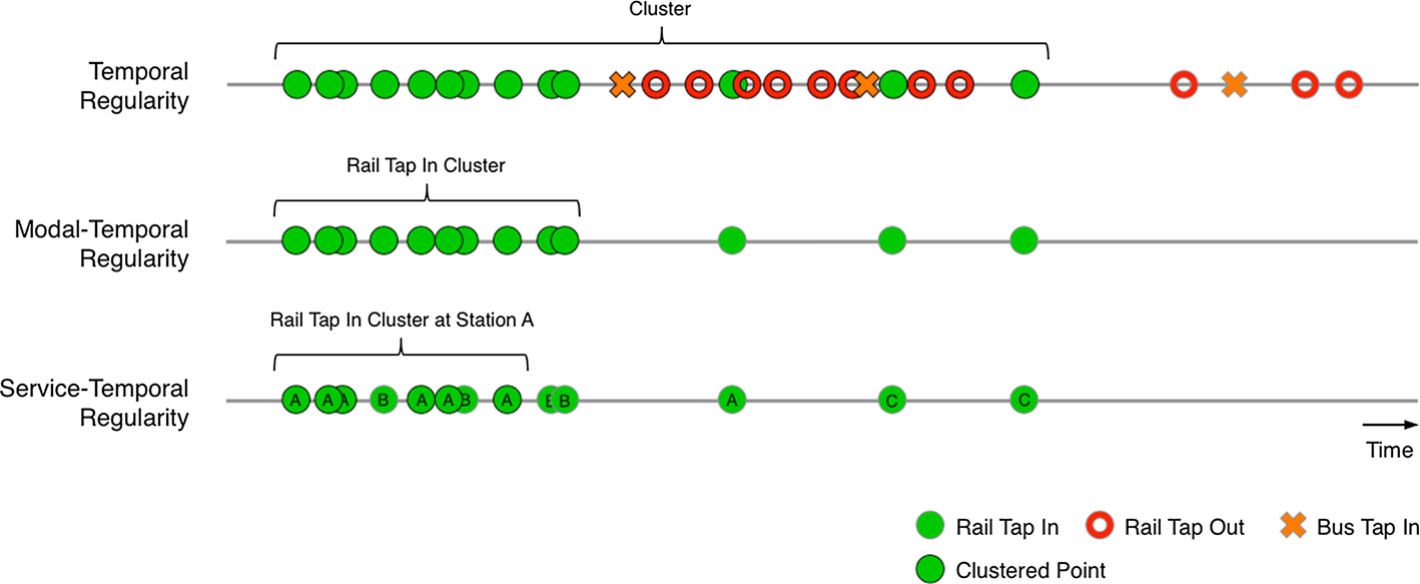
DBSCAN cluster formation at different scales, each cluster meets the conditions defined by the *dist* and *minPts* parameters

Figure 1 explains how clusters are formed using DBSCAN through use of each subset of the data. As can be seen, clusters are formed according to a threshold of point density, which varies according to the data incorporated. By including all transactions, a larger cluster is formed, spanning a wide timespan. Greater specificity in the subset of the data—either by mode or service—naturally leads to a reduced cluster size. In all instances the DBSCAN parameters govern the formation of clusters, excluding any points outside of the *dist* threshold, and clusters of points not exceeding the *minPts* parameter (most clearly seen through absence of cluster of Station B points in the Service-Temporal example). Readers are referred to the paper by Ester et al. (1996).

Regularity clusters will be generated from the trip records of each individual traveller. By extracting regularity at the individual scale, it will be possible to aggregate across spatial and temporal definitions, providing a detailed view of regularity across the transportation network. It will furthermore enable the extraction of linked regularity clusters, highlighting where interdependencies between different types of travel behaviour exist. Details of how regular interactions are determined will be provided below.

## Regularity at different scales

The first stage of our analysis examines the nature of regularity across each or our three definitions Having derived regularity clusters for each traveller for each definition, this analysis will describe their appearance, structure, and persistence over time. Following the description of each form of regularity cluster, we will then describe interactions between cluster types.

### Temporal regularity

The first stage of analysis uses definitions of regularity drawn from all travel transactions, regardless of mode or service. Across the entire dataset, 4.78 m users are identified to have at least one cluster of regular activity during the study period, resulting in 454 m regular journeys. On average, users have 2.50 regular cluster periods each, breaking down to just over 1.01 m users (roughly 21%) with a single regular cluster, 1.8 m users (38%) with two clusters, 1.05 m users (22%) with three clusters, 566,611 users (12%) with four clusters, and the remaining 355,884 users (8 %) with five or more regular clusters.

**Fig. 2:**
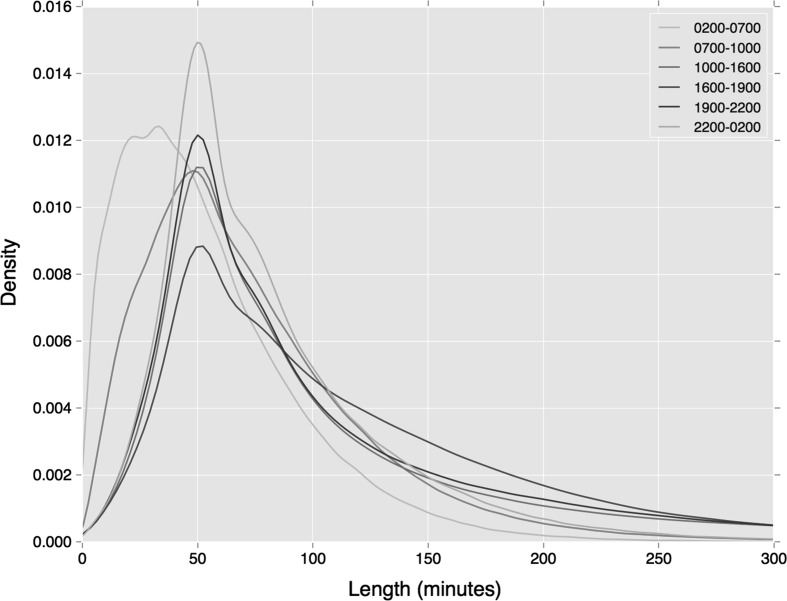
Distribution of cluster length by period of departure

The temporal extent of the cluster provides insights into the variance in travel times captured within the cluster. An average temporal extent of 112 min is found across all clusters, incorporating the entire travel period (e.g. from ‘tap-in’ to ‘tap-out’) plus all variances around these times. Deeper insight is gained by breaking these trends down by time of day. As can be seen in Fig. 2,^1^ proportionately shorter mean temporal extents in regular behaviour are found during the early pre-morning (2–7 am) and morning peak (7–10 am) periods. This indicates that individuals travel with slightly lower variance in departure times during earlier periods.

**Fig. 3:**
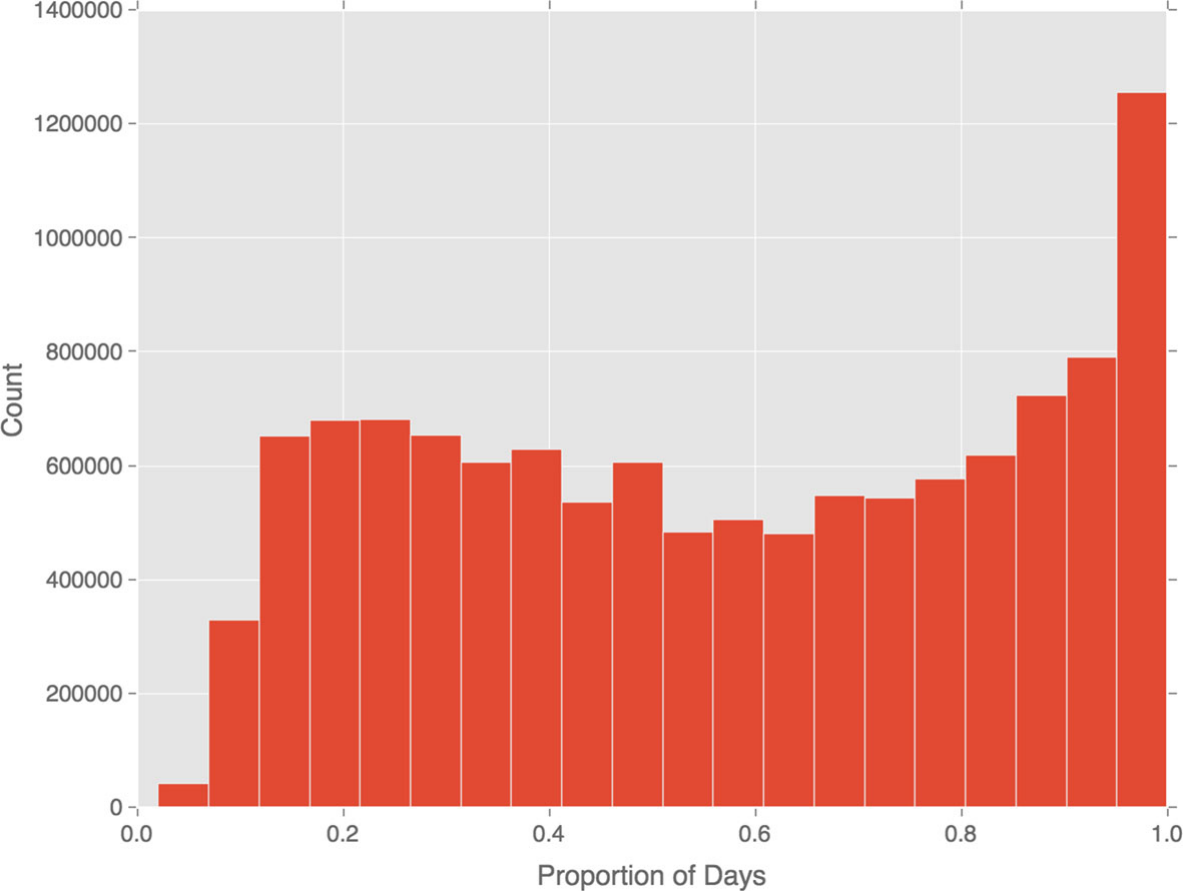
Proportion of days travelled that clusters are observed

Figure 3 provides an insight into the persistence of regular behaviours, showing how often clustered travel behaviour is observed relative to all of the days on which the individual travels. Over 120,000 clusters are observed on every day that the individual travels, indicating a large portion of travellers with a very strong attachment to this regularity cluster. Yet below this level an even spread is observed, suggesting the persistent usage of these clusters varies widely.

### Modal-temporal regularity

The second stage of our regularity analyses focus on the use of particular modes of transportation. Due to the definitions in the Oyster card dataset, we are able to build regularity clusters that differentiate between rail and bus travel, providing insights into the differences and similarities in regular travel on each mode. Looking first at regularity on the Underground and rail network (henceforth referred to as ‘rail’ travel for brevity), 2.434 m users exhibit temporal clusters in trip start (i.e. ‘tap-in’) activity where there is an average of 2.3 clusters for each individual. This results in the classification of 112 m journeys as being regular. Across all users, 564,797 (roughly 23%) exhibit only one cluster of activity, 1.13 m (47%) have two clusters, 442,268 users (18%) have three clusters, and 293,172 (12%) have four or more clusters. These trends would appear to align with the expected predominant home-to-work travel patterns. These clusters have an average duration of 77 min, which is shorter than the temporal regularity clusters described above. This is likely due to these not incorporating travel times (e.g. ‘tap-in’ and ‘tap-out’ actions are separated). Near identical trends on all measures are identified with respect to trip end (i.e. ‘tap-out’) activity.

The same measures can be drawn out for regular travel by bus. Buses have a higher number of regular users than rail, with 3.22 m regular travelling individuals who take 207 m journeys. Like regular rail travel, users exhibit an average of 2.3 clusters each. However, this breaks down somewhat differently with a greater proportion of travellers regularly using the bus over a single period (28.5%, or 917,511 users), a considerably lower proportion travelling by bus over two periods (35%, or 1.14 m users), and higher proportions using the bus over four periods or more. Bus regularity deviates from rail in terms of departure times too. The duration of bus clusters average 110 min in length, considerably higher than across rail modes.

**Fig. 4:**
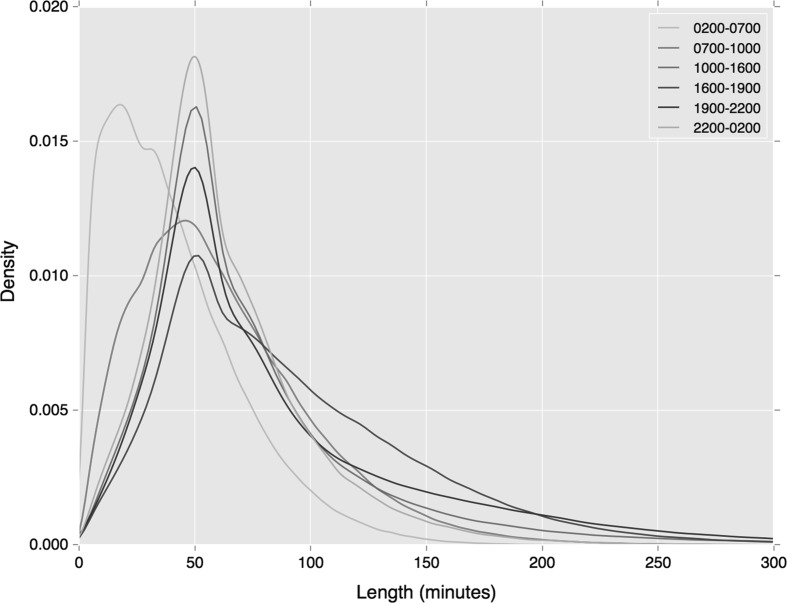
Distribution of rail tap-in cluster lengths by period of departure

**Fig. 5:**
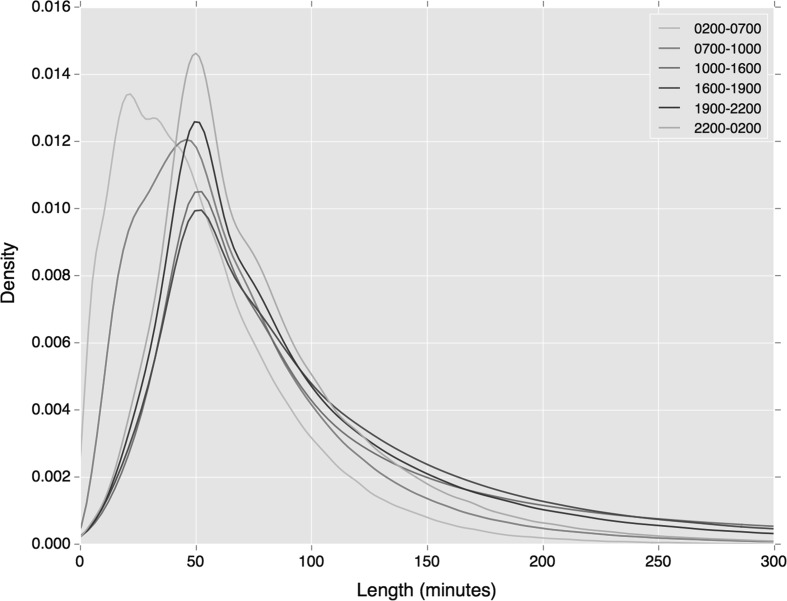
Distribution of bus cluster lengths by period of departure

Figures 4 and 5 show distributions of cluster length by time period of both regular rail and bus travel. Both broadly reflect the wider temporal trends shown in Fig. 2, where early pre-morning and morning peak travel indicates lower variance in departure time. However, these trends appear starker in the case of rail travel, where a higher density of users exhibits a short variation in regular travel behaviour.

**Fig. 6:**
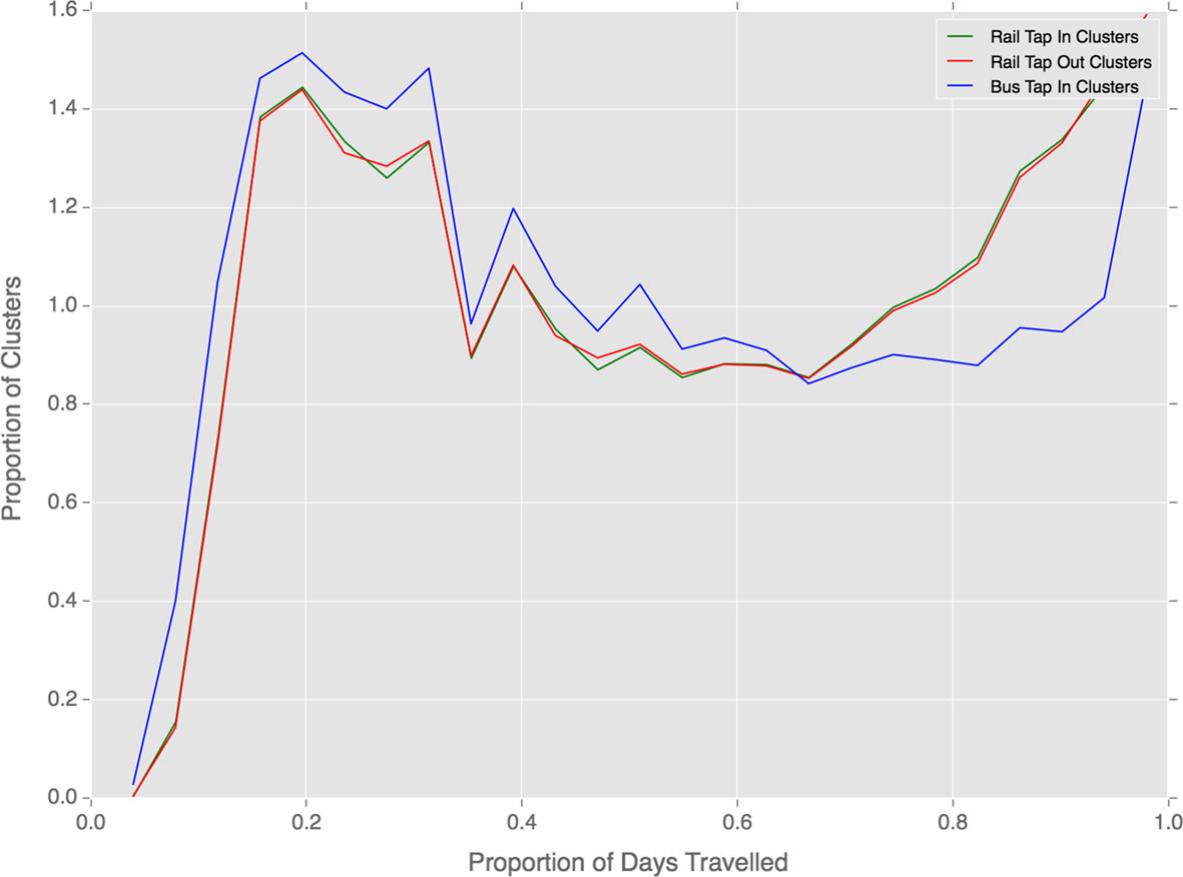
Proportion of days travelled that clusters are observed split by travel mode

Additional minor deviations in regular use of each travel mode are observed in the persistence of clusters over time as shown in Fig. 6. While both follow the broad trends exhibited across all temporal regularities (Fig. 3), regular bus travel is more concentrated in lower proportions of days travelled. This means travellers are using the bus at a more regular times, but relative to rail travel they are not using the service as often on a daily basis. Rail travel thus shows a greater proportion of individuals travelling at a regular time almost every day on which they travel.

### Service-temporal regularity

The final set of indicators associated with our third definition of regularity is associated with specific rail stations and bus services. This definition sets a much stricter specification on regularity than previously. By incorporating the specific station within the regularity definition, it is found that the number of regular travellers by rail (using ‘tap-in’ activity) reduces to about 2 m individuals, constituting 80 m trips. These travellers have an average of 2.4 rail clusters, broken down as 504,258 (or 25%) with only one cluster, 815,299 (41%) with two clusters, and 336,267 (17%) with three clusters of activity. These distributions align with the more general definition of regular rail travel offered earlier. However, comparatively, a reduction in the variance of departure times is observed, with mean cluster length dropping to 54 min. Once more, similar trends in ‘tap-out’ behaviours are found.

Classification of regular bus travel requires a clear association with a specific bus route. Given that some bus routes overlap, this leads to a stricter definition of regularity than the definitions associated with rail stations above. The clustering process results in regularity clusters for 2.2 m individuals taking a total of 30.6 m journeys, reduced from the earlier bus regularity measures, but with an increased average number of clusters, which rises to 3.3. Unlike the earlier measures, the highest proportion of travellers have only one cluster (612,274 or 28%), with 556,908 (25%) having two clusters, 317,395 (14%) with three clusters, and 720,853 (33%) with four or more clusters. The greater distribution of travellers with a high number of clusters is indicative of regular bus travellers using multiple bus routes, possibly from a bus stop served by replicate services. The mean cluster length is considerably shorter again than the more general measure at 54 min.

**Fig. 7:**
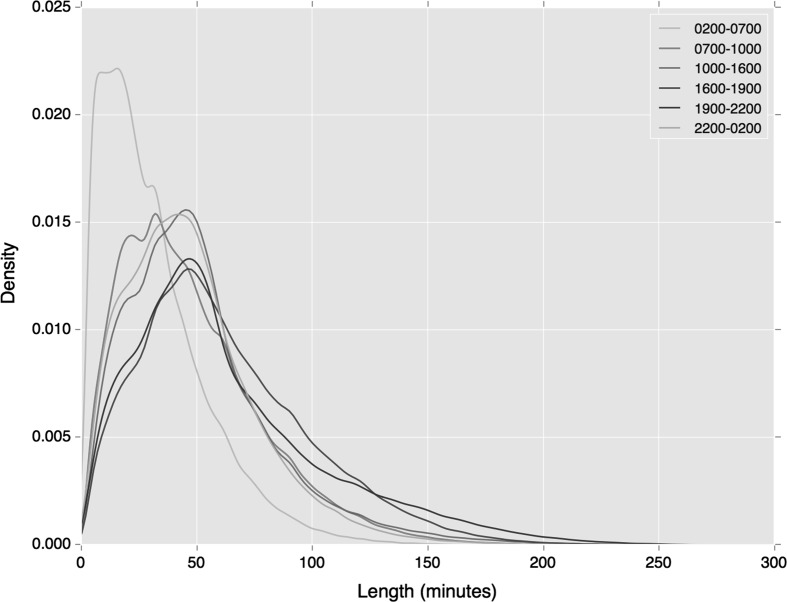
Distribution of rail tap in cluster lengths, associated with a specific station by period of departure

**Fig. 8:**
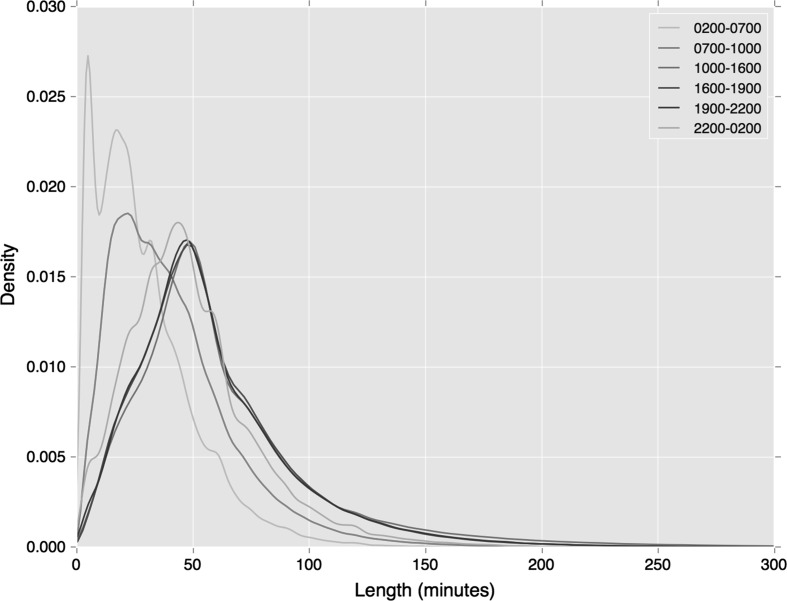
Distribution of bus tap in cluster lengths, associated with a specific station by period of departure

The distribution of cluster length by time period again demonstrates how the greater specificity of the clustering measure leads to reduced variation in departure time. As shown in Figs. 7 and 8, compared against our earlier similar analyses (see Figs. 4 and 5), variations in departure time reduce. This is most noticeable during the early morning period on both travel modes, and the morning peak period during regular bus travel.

**Fig. 9:**
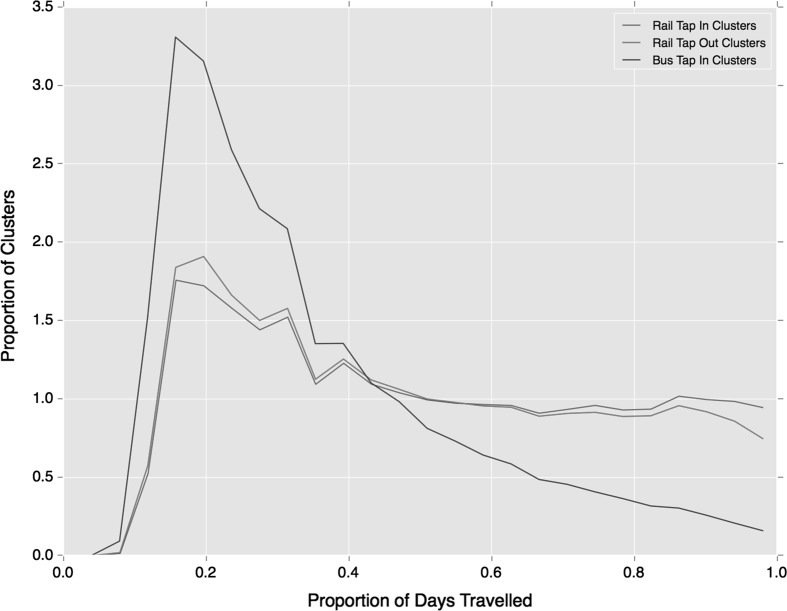
Proportion of days travelled that clusters are observed split by travel mode, using service-temporal clustering

Moving to the persistent appearance of clusters over days travelled by the individual, shown in Fig. 9, one observes a general reduction in the frequency of clusters being observed every day, relative to earlier trends (Figs. 3 and 6). Furthermore, a greater discrepancy between bus and rail travel is found with very few regular bus travellers regularly using the same bus service on more than 0.8 of their days travelled. This may be indicative of the availability of multiple bus services at some bus stops. Rail travel maintains a similar trend to previous clustering results, although this presents a lower everyday usage.

By considering three distinct but related definitions of regularity, it has been possible to establish how each definition leads to extracting a different type of behaviour. As seen in the final section where the definition of regularity becomes more specifically associated with spatial locations, temporal variation within the cluster reduces, bringing these behaviours closely with peak travel times. Where looser definitions of regularity are employed, the temporal distribution of regular travel becomes more varied, and suggests that while inter-peak travel may be regular, it is also less spatially and temporally specific. This insight has implications for how we understand and analyse regularity, but also allows us to potentially identify subsets of regular travellers and their flexibility to change in their usual travel mode or route.

### Regular cluster interactions

Beyond patterns in singular modal regularities, the approach also enables us to explore regular interactions between activity clusters. This stage involves identifying instances where regular travel behaviours (e.g. points that have been clustered) interact. To do so, all instances where two clustered points fall within 30 min of each other are logged as an interaction between regular clusters. The 30-min window allows for walking between train stations and bus stops, and any subsequent waiting times. A *regular interaction* is then only recorded where an interaction occurs on over 50% of the occurrences of the first cluster. For instance, if a cluster of bus activity is observed on 60 occasions, a regular interaction with a rail cluster will only be formed if points intersect within 30 min on more than 30 occasions.

Taking modal-temporal regularity initially, by these definitions of regular interaction, we observe that there are 12.4 m interactions over 582,385 pairs of bus and rail (‘tap-in’) clusters. This means that 6% of regular bus trips lead to a regular rail journey. In reverse, there are 13.46 m interactions between 689,803 pairs of rail (‘tap-out’) and bus clusters, meaning 12% of regular rail journeys lead to regular bus travel. Moving to service-temporal regularity, a total of 8.2 m interactions between regular bus and rail (‘tap-in’) are identified across 702,178 pairs of clusters. This means 27% of all service-specific regular bus journeys lead to a regular journey from a specific station, a large increase on the equivalent measures noted above. In reverse, from rail (‘tap-out’) to bus cluster, interactions drop to 3.87 m instances across 301,737 pairs of clusters, which relates to only 5% of all regular rail travel leads to a regular travel on a specific bus service.

The discrepancies in these trends are likely to be influenced by the highly regular work-based travel that is predominant using this definition of regularity. These trends indicate that there are areas of significant dependency between bus and rail across the transportation network. Expanding further, it is possible identify the most important points of regular interaction between travel modes. Tables 2 and 3 shows the top ten interactions in both directions. All stations listed are on the Underground network in primarily residential regions of London, well served by the bus network. It is interesting to note that, although some pairs appear in both directions, there is variation in order and magnitude. Deviations in the number of regular interactions are indicative of the same trends observed above.

**Table 2:** Strongest regular connections between specific bus routes and rail stations, using service-temporal clustering

Bus route	Rail station	Total regular interactions
472	North Greenwich	78,849
W7	Finsbury Park	68,207
5	Barking	60,848
86	Stratford	59,102
486	North Greenwich	42,752
W3	Finsbury Park	39,413
257	Stratford	36,354
207	Shepherd’s Bush	34,583
158	Blackhorse Road	34,421
97	Walthams to w Central	32,748

**Table 3:** Strongest regular connections between specific rail stations and bus routes, using service-temporal clustering

Rail station	Bus route	Total regular interactions
Finsbury Park	W7	45,161
North Greenwich	472	41,843
Barking	5	32,541
Victoria	38	25,607
Finsbury Park	W3	25,268
North Greenwich	486	25,053
Bethnal Green	8	24,679
Waterloo	521	24,530
Whitechapel	25	22,421
Blackhorse Road	158	20,346

## Spatiotemporal variation in regularity and irregularity

While trends in regularity provide some insight into travel behaviour, regular behaviours can be better understood relative to all travel. The intention of the second stage of this analysis is to therefore provide insights into the persistence of regular travel relative to irregular, ad hoc travel. These insights should provide a view of the type of regular behaviour unfolding at different places and times across the transport system. There are three major concerns underlying this phase of the analysis, first to establish the nature and degree of regularity across different transport modes, second to observe how regularity varies across space and location, and third to explore how regularity varies across different time periods. In each case, the volume of regular trips, defined using the above approaches, are measured against all comparable trips.

### Modal and service regularity and irregularity

In this stage of analysis, we explore how each definition of regularity compares against all comparable trips taken. These values are calculated using the counts of trips classified as regular, using each type of regularity clustering, against counts of all trips taken (shown in Table 1). A summary of the results is given in Table 4.

**Table 4:** Proportion of trips classified as regular using each form of regularity clustering

Transaction type	Regularity classifier	Total regular trips	Proportion of regular trips
All clustered activity	Temporal	453,880,547	0.71
In Underground tap-in cluster	Modal temporal Rail Tap-in	112,014,214	0.62
In Underground tap-in cluster with location	Service-temporal Rail Tap-in	79,723,394	0.44
In Underground tap-out cluster	Modal Temporal Rail Tap-out	110,610,167	0.61
In Underground tap-out cluster with location	Service-temporal Rail Tap-out	76,236,825	0.42
In Bus tap-in cluster	Modal temporal Bus Tap-in	207,351,032	0.75
In Bus tap-in cluster with service	Service-temporal Bus Tap-in	30,627,664	0.11

The first type of regularity examined is Temporal Regularity, where trips are clustered using their transaction time only. A count of all temporally clustered trips is extracted for all users (45.39 m transactions), and compared against all transactions (639.98 m). The results show that 0.71 of trips can be considered clustered in time at the individual traveller level. This value provides a benchmark for analysis of the modal and service-specific clustering approaches. Moving to Modal-Temporal regularity, some clear deviations around the benchmark can be found. Bus trips are shown to be more likely to be regular, with a proportion of regularity at 0.75. ‘Tap-in’ and ‘tap-out’ event regularity proportions are similar, but show lower regularity than bus trips, at 0.62 and 0.61 respectively. This indicates a higher proportion of irregular journeys being undertaken on the Underground than on the bus network.

This exploration can be broken down further by considering regularity as attached to a specific station or service. In this case we see a reduction in the proportion of Underground trips that may be considered regular, relative to travel using any station. Nevertheless, the proportions of 0.44 for ‘tap-in’ events and 0.42 for ‘tap-outs’ suggest a reasonably strong, continuing association between individuals and specific locations, indicating that nearly half of all trips to or from any Underground station are undertaken regularly. The results furthermore suggest a small indication of greater irregularity in destination choice (‘tap-out’ location) than in origin choice.

These proportions are considerably lower for bus routes, however. Only 0.11 of all bus trips are regularly associated with a specific service, considerably lower than the 0.75 proportion indicated for regular travel via any bus service. Rather than indicating a degree of randomness in bus route selection, these results may be indicative of redundancy in bus routing. At major convergences in bus routes, many individuals have a choice of two or more routes to reach their destination, meaning no single route is classified as a regular association.

### Station and service-specific regularity and irregularity

Taking this further slightly, one can explore which specific stations and bus services exhibit high proportions of regularity. This definition provides potential insight into the land use functions around the station (e.g. as a commuter hub, concert venue, etc.), or the types of travellers that may be using it (e.g. regular commuters, tourists, etc.). In this analysis, the service-temporal regularity definitions are used to establish a proportion of regular trips occurring at each station or on the relevant bus services.

By way of explaining these trends, we have explored how these trends relate to underlying residential and workplace population distributions. Population data is drawn from the 2011 UK Census, and indications of residential and workplace populations are calculated as the population density within a 2 km buffer radius around the station location. These methods lead us to mean population density measures of 89,787 (max 186,860, min 2379) people per square kilometre (ppkm) for residential populations, and 94186 ppkm (max 748,307, min 893.2) for workplace populations at each station. These measures will provide indication of the predominant land use function (i.e. workplace or residential) of the land surrounding each station. While spatial patterns in regularity are undoubtedly impacted by the land use and demographic characteristics of the region, a full analysis of these trends exceeds the purview of this paper and is recommended as further work.

**Fig. 10:**
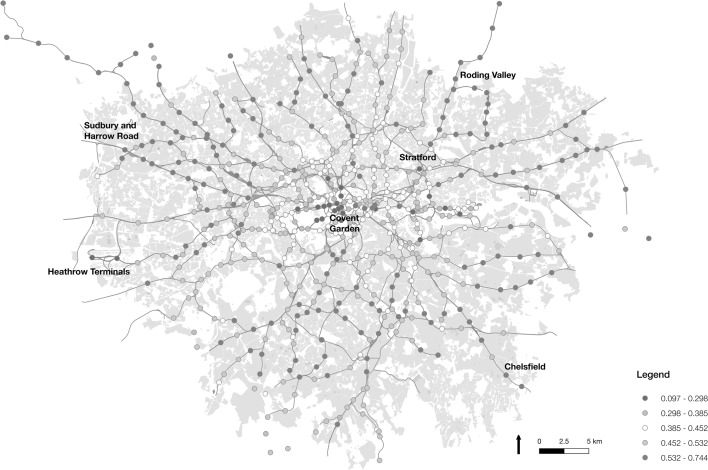
Map of underground and rail stations in London visualised by the proportion of regular trips originating at each location

Examining Underground and rail origin stations first, as demonstrated in Fig. 10, there are clear spatial differences in the proportion of regular trips occurring at different stations. For the most part, higher proportions of regular trips are found in outer London, with lower regularity at origins in central London. The area of highest consistent regularity appears to be the northwest area of London. Looking specifically at stations, the greatest proportion of regular trips are found originating at the Sudbury and Harrow Road station, where 0.84 of trips are classified regular, considerably higher than the 0.44 mean across all stations. Other stations with a high ranking include Roding Valley (0.70), Northolt Park (0.68) and Chelsfield (0.67), and are all similarly located in outer London. Relative to population distributions, we observe that the residential population densities of these stations fall someway below the London mean at 49402 ppkm, suggesting relatively low residential population densities. However, workplace populations are much lower, at 15077 ppkm across these stations, showing they are not strongly associated with workplace locations.

At the other end of the scale, the least regular, or most irregular, origin station is Heathrow Terminal 4, with a regularity proportion of 0.07. The other Heathrow airport terminal stations are placed fourth lowest (Terminal 5 with 0.17) and eighth lowest (Terminals 1, 2 and 3 on 0.19), while City Airport also ranks low (seventh on 0.19). Also ranking low in terms of regularity are central London locations in and around the West End. These include Covent Garden (third lowest on 0.16), Leicester Square (sixth on 0.19), Bayswater (ninth on 0.20), Hyde Park Corner (eleventh on 0.22), and Piccadilly Circus (twelfth on 0.22). The trends at each of these stations may be attributed to the relatively high volumes of tourists staying in these locations, compared to commuters with more regular travel patterns.

**Fig. 11:**
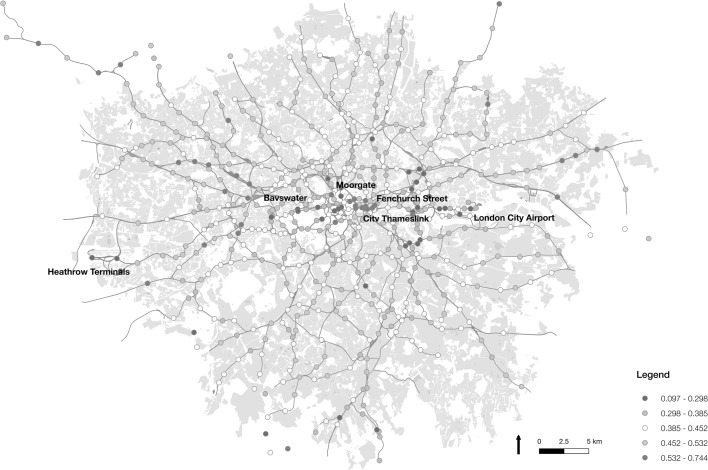
Map of underground and rail stations in London visualised by the proportion of regular trips ending at each location

Moving to regularity at destinations as shown in Fig. 11, a more mixed spatial distribution is observed. The majority of the highest-ranking destinations are made up of locations in or near to the City of London, the major financial district in the city. This provides some indication of the more formal working procedures in this area relative to the rest of the city. The stations around the City area include Fenchurch Street (ranked first with 0.74), City Thameslink (second with 0.73), Moorgate (fourth with 0.64), Cannon Street (fifth on 0.62), Mansion House (sixth on 0.61), Farringdon (eighth on 0.60) and Bank (ninth on 0.60). These regions exhibit very high levels of workplace population too, averaging 663,592 ppkm, and only near average residential population density (119,874 ppkm). However, outer London locations too, such as Sudbury and Harrow Road (0.71) and Croxley (0.58), rank high again, reconfirming the strong regularity associated with travellers to and from these particular stations. These locations are conversely associated with very low workplace population densities (25,494 and 11,132 ppkm respectively). This highlights the presence of a mixed relationship between regularity and the distribution of populations, and indicative of there being multiple different forces may be at play. In the cases of stations in the City of London, this may be linked to workplace density, but in the cases of outer London stations, this must be more associated with demographics and service availability.

At the other end of the scale, many of the same locations are found as highly irregular destinations as were identified as irregular origins. Heathrow Terminal 4 again ranks lowest with 0.1, with other airport terminals and tourist locations showing low proportions of regular trips.

**Fig. 12:**
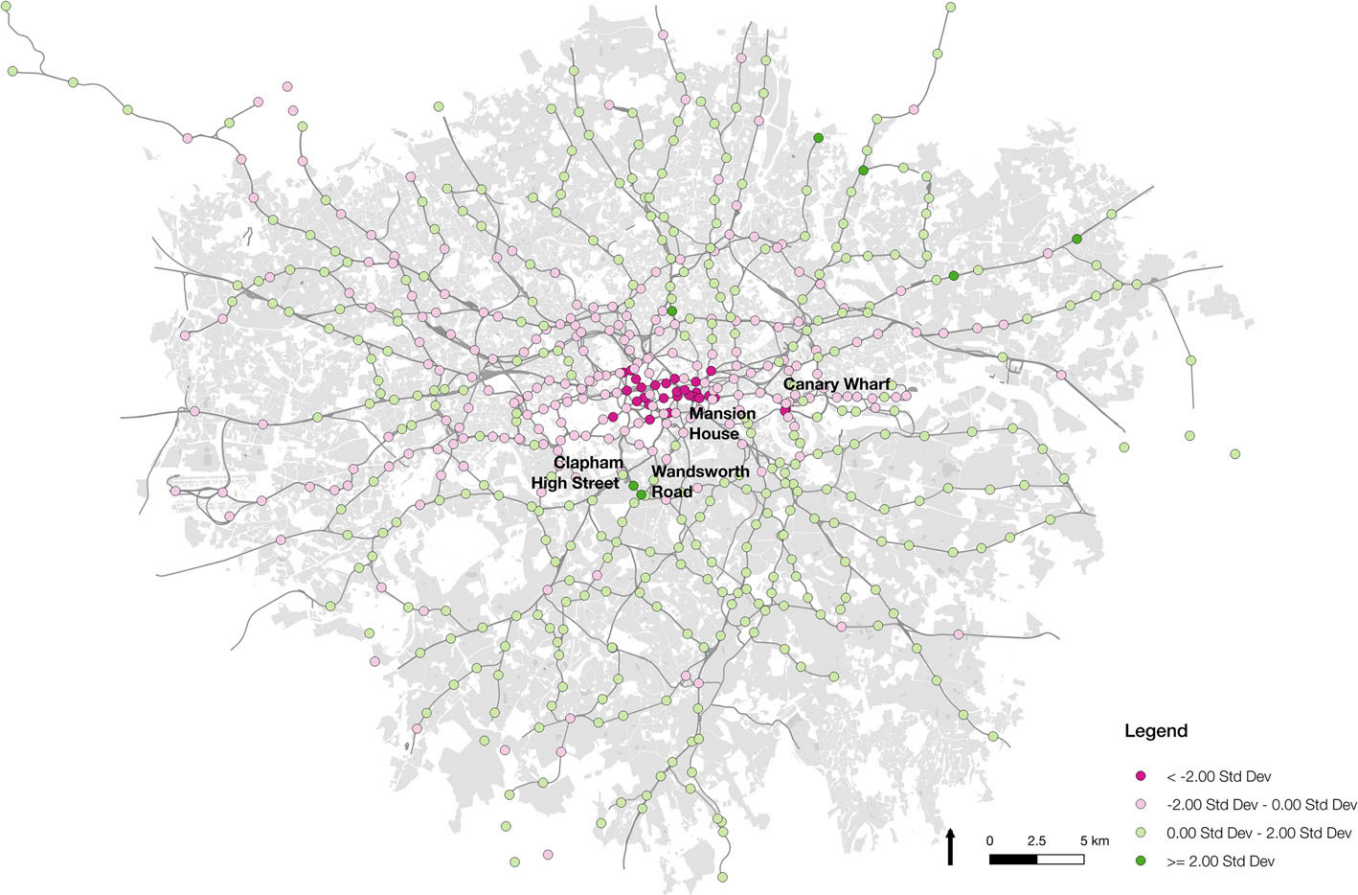
Map of underground and rail stations in London visualised by the differences in proportion of regular trips starting and ending at each location

Examining the difference between proportions of regular origin and regular destination trips, it is possible to examine more deeply the dominant roles of each station. A difference value is achieved for each station by subtracting the proportion of originating trips classified as regular from its equivalent proportions as a destination. As shown in Fig. 12, there are clear spatial trends in the differences across all stations. With largely negative values, the map shows central London as a source for a greater proportion of regular trip destinations than regular origin journeys. These patterns furthermore spread east to the Canary Wharf financial district. Within these areas, Mansion House (a −0.17 difference), Canary Wharf (−0.15), St Pauls (−0.14), and City Thameslink (−0.14) show the greatest differences. The reason for these differences may be two-fold. Trips to these locations may be strongly associated with morning work commuter trips, meaning a stronger time constraint on the time taken to travel. These locations exhibit much higher than average workplace populations, and so may also see more irregular journeys for purposes of meetings and other business activity, leading to an increase in irregular journeys relative to regular trips.

Certain locations, such as Wandsworth Road (+0.28), Clapham High Street (+0.24) and Gidea Park (+0.24) show significantly higher proportions of regular origin journeys than they do destination journeys. This may indicate a greater heterogeneity in the time of journeys to these destinations, or highlight the role of other nearby stations as alternative destinations (but not as viable origins). This question requires further exploration than is possible from these data alone. As discussed earlier, there is a lower association between regular travel and specific bus routes where the proportion across all stations is 0.11. Many of those routes with relatively high levels of regularity appear to originate or end at Underground or rail stations (including routes ‘346’ with a 0.31 regularity proportion, ‘W7’ with 0.3, ‘521’ with 0.29, and ‘209’ with 0.27). Although the location of bus boarding cannot be confirmed, this trend suggests a strong interconnectivity between rail and bus at these locations. The lowest ranking routes for regularity were found to be those routes designated for school travel. The reason for this may be the time of year that the study was undertaken (during school holidays).

### Temporal patterns of regularity and irregularity

In this second stage of analysis, we will also explore how regularity and irregularity vary over different periods. This work will be divided into finer scale variations over the course of a weekday, variation over different days of the week, and variation over the whole two-month study period. In each case, variation is explored for each definition of regularity with the aim of drawing out additional insights.

#### Time of day variation

Variation over the course of the day provides further context around the nature of regularity discussed in “Regularity at different scales” section. In keeping with these analyses, results are presented both across discrete time periods, and by assessing continuous variation over the course of the day. The summary results by time period are presented in Table 5.

**Table 5:** Proportion of trips classified as regular using each form of regularity clustering, broken down by time period

Classification	Early	AM peak	Inter	PM peak	Evening	Late
All activity	0.81	0.79	0.64	0.78	0.66	0.50
Underground tap-in	0.83	0.82	0.37	0.71	0.52	0.35
Underground tap-in with location	0.76	0.73	0.17	0.49	0.27	0.17
Underground tap-out	0.80	**0.85**	0.37	0.68	0.56	0.33
Underground tap-out with location	0.71	0.73	0.16	0.43	0.34	0.21
Bus	0.84	0.84	0.73	0.77	0.68	0.57
Bus with service	0.27	0.21	**0.04**	0.12	0.10	0.08

Maximum and minimum values are highlighted in bold

**Fig. 13:**
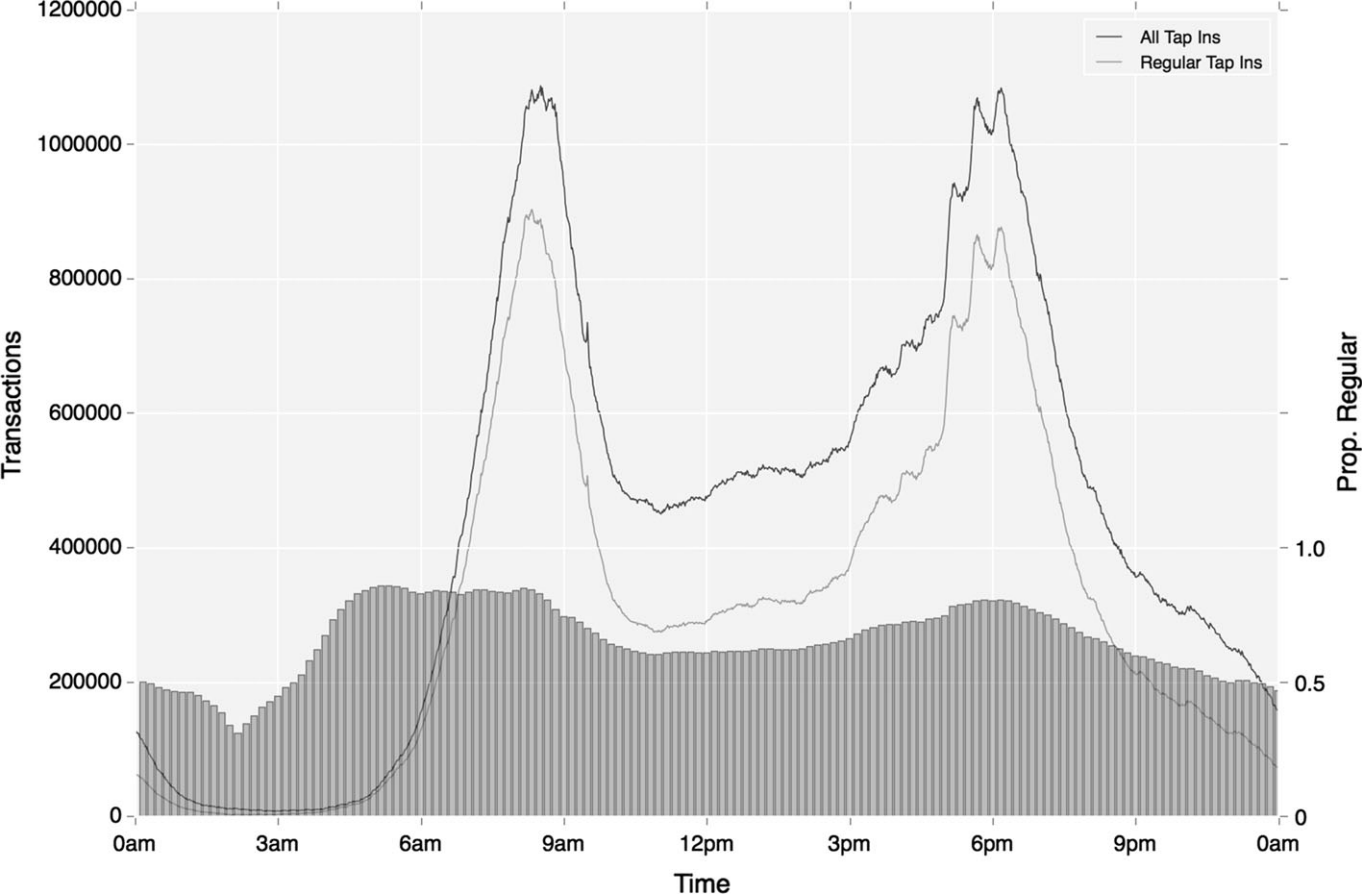
Temporal variation in regular travel activity, shown in absolute (line graph, left axis) and relative (bar graph, right axis) terms against all travel (same graph format is applied in Figs. 14–19)

Looking at all activity in the first instance, the results in Table 5 suggest there is clearly some variation in regular activity during the course of the day. The highest degree of regular activity is seen during the early period, where 0.81 of all travel is classed as regular. This is mostly likely associated with high volumes of commuter travel relative to more ad hoc behaviours. Peak travel is furthermore observed to be highly regular too, with 0.79 and 0.78 proportions of activity considered regular during morning and afternoon periods respectively. The highest degree of irregularity is found in the late period, suggesting a stronger association with ad hoc activities at these times (e.g. social meetings). Figure 13, provides greater detail around the evolution of these patterns over time, and demonstrates how regularity increases at a lower rate than the increases in trip volume at peak time.

**Fig. 14:**
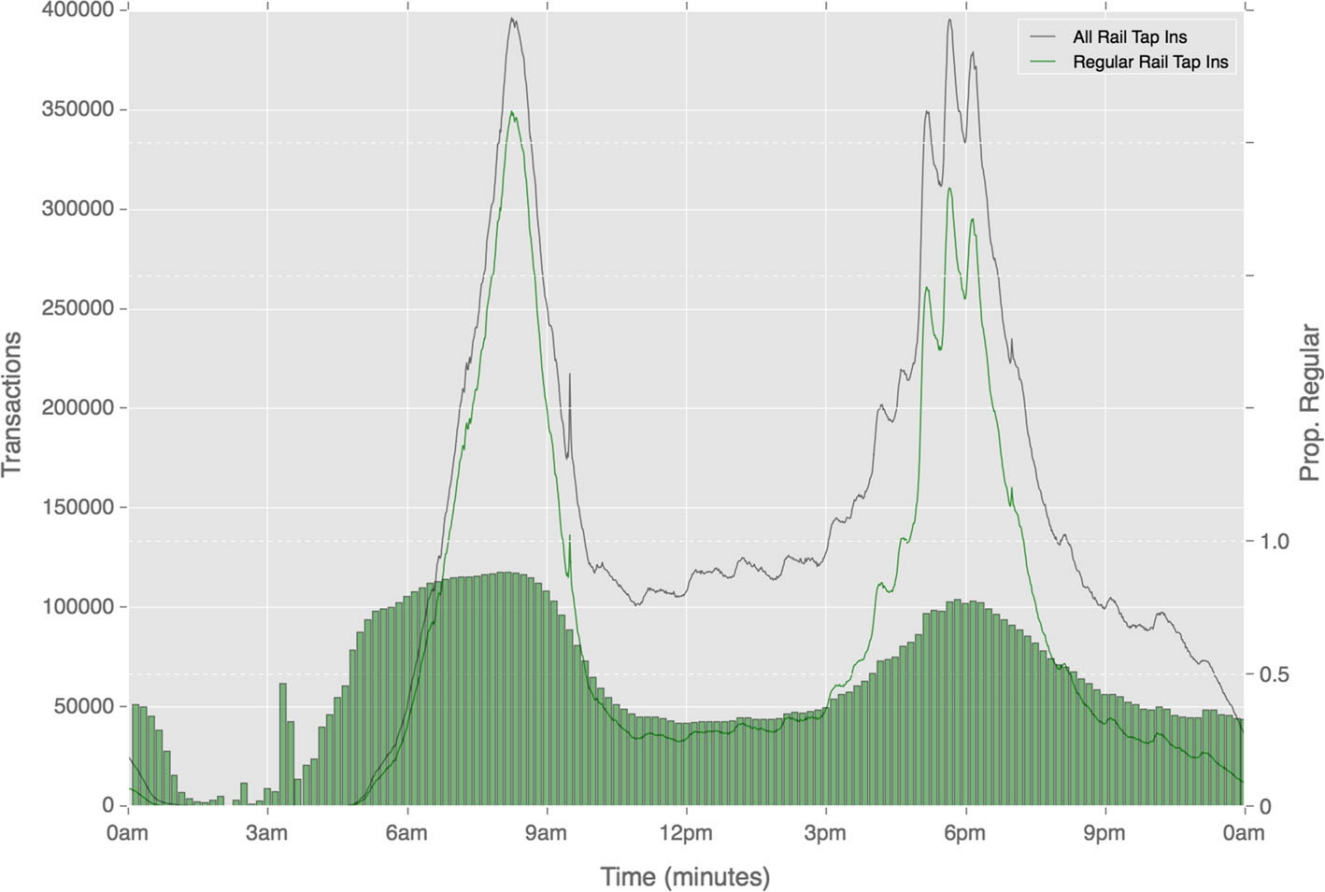
Temporal variation in regular ‘tap in’ rail activity (absolute and relative), based on mode choice alone

Breaking down results by travel mode, again regularity is shown to increase during peak time, however the variation across time periods is considerably more striking. Taking ‘tap-in’ activity on to the Underground and rail networks, while travel during early and morning peak on these modes is very high, this reduces to around 0.35 during Inter and Late period time, a much more significant jump than across all travel. Figure 13 provides more detail on these trends, and highlights the starker variation in regularity proportions over the course of the day. It is also interesting to note that small perturbations in regular rail activity are revealed at regular intervals (most notably shortly after 17:00, 17:30, 18:00 and 20:00, but also throughout the inter-peak period). These times would appear to follow typical workplace closing times. Regularity proportions closely follow these small peaks, so providing further evidence of these trips representing work-related travel.

**Fig. 15:**
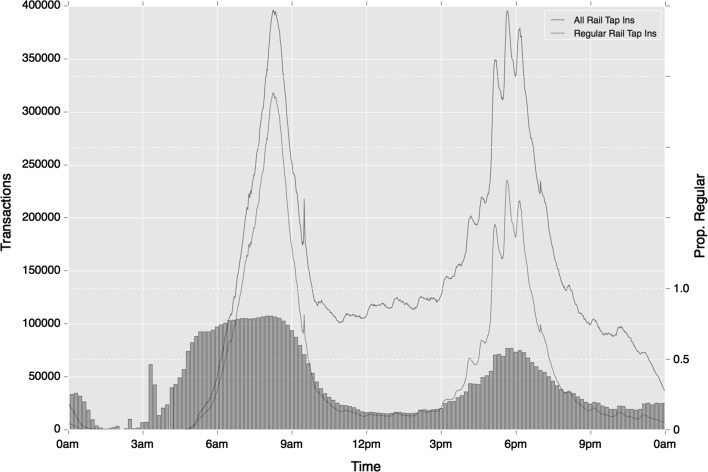
Temporal variation in regular ‘tap in’ rail activity (absolute and relative), based on mode and origin location choice

The variations across time become more severe once origin location is incorporated into regularity. Strong regularity is observed during morning periods which drops substantially during the rest of the day to 0.17 during the Inter period. This reduction is considerably starker than mode-temporal regularity alone, as demonstrated where comparing Fig. 15 with Fig. 14. One additional trend of note are the strong differences in the degree of regularity between morning and afternoon peak periods. The latter period exhibits a reduced level of travel regularity, indicating greater flexibility and variation in evening travel. Although a similar volume of trips take place during each period, these trips are less regular. According to mode-temporal regularity measures, a 13% decrease in regularity is observed between the two periods. This reduction, however, jumps to 33% where the origin location is included in cluster calculation. These trends do not only reflect greater flexibility in the nature of travel in the afternoon peak for they also highlight greater variation around the specific origin of travel during this period.

**Fig. 16:**
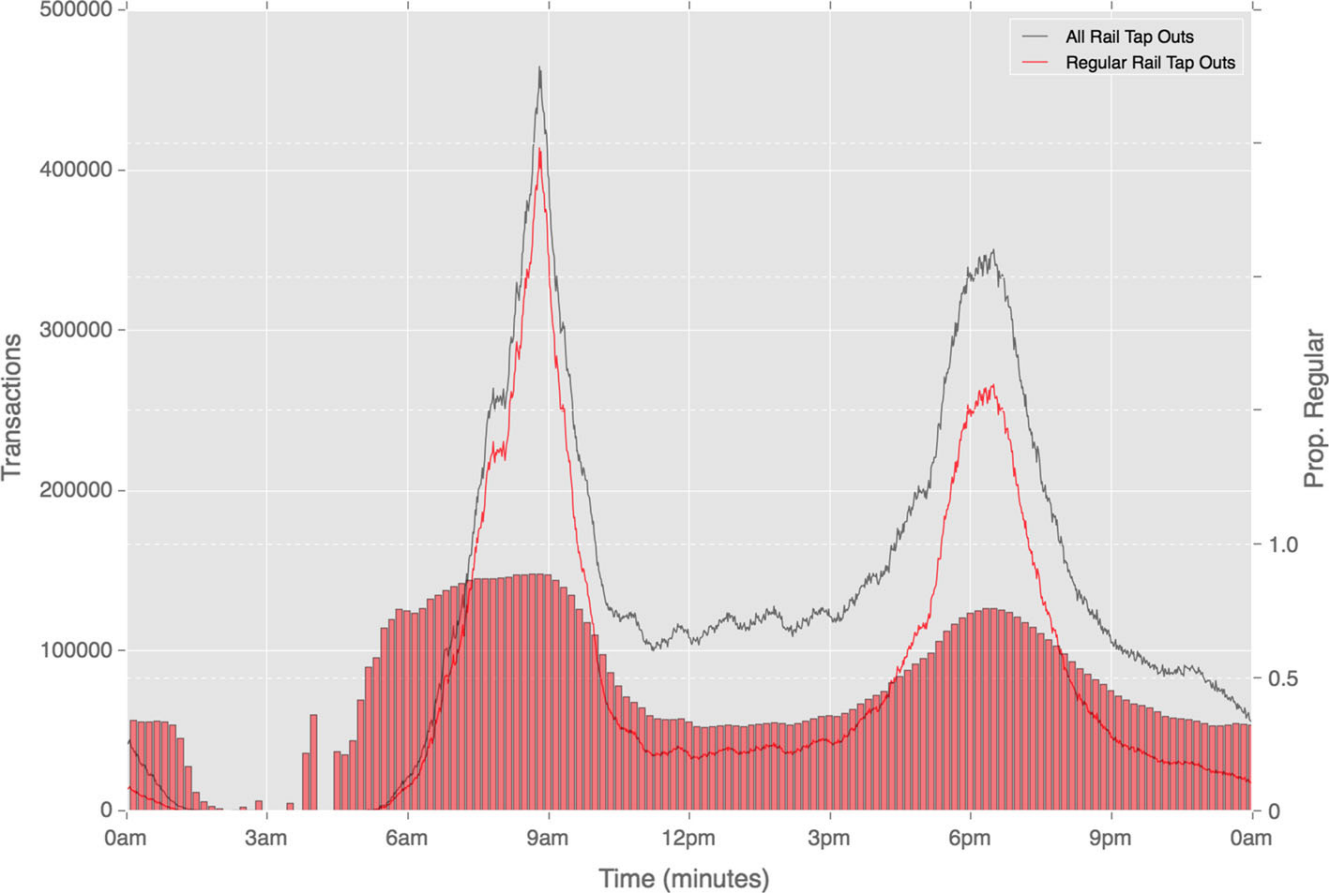
Temporal variation in regular ‘tap-out’ rail activity (absolute and relative), based on mode choice alone

**Fig. 17:**
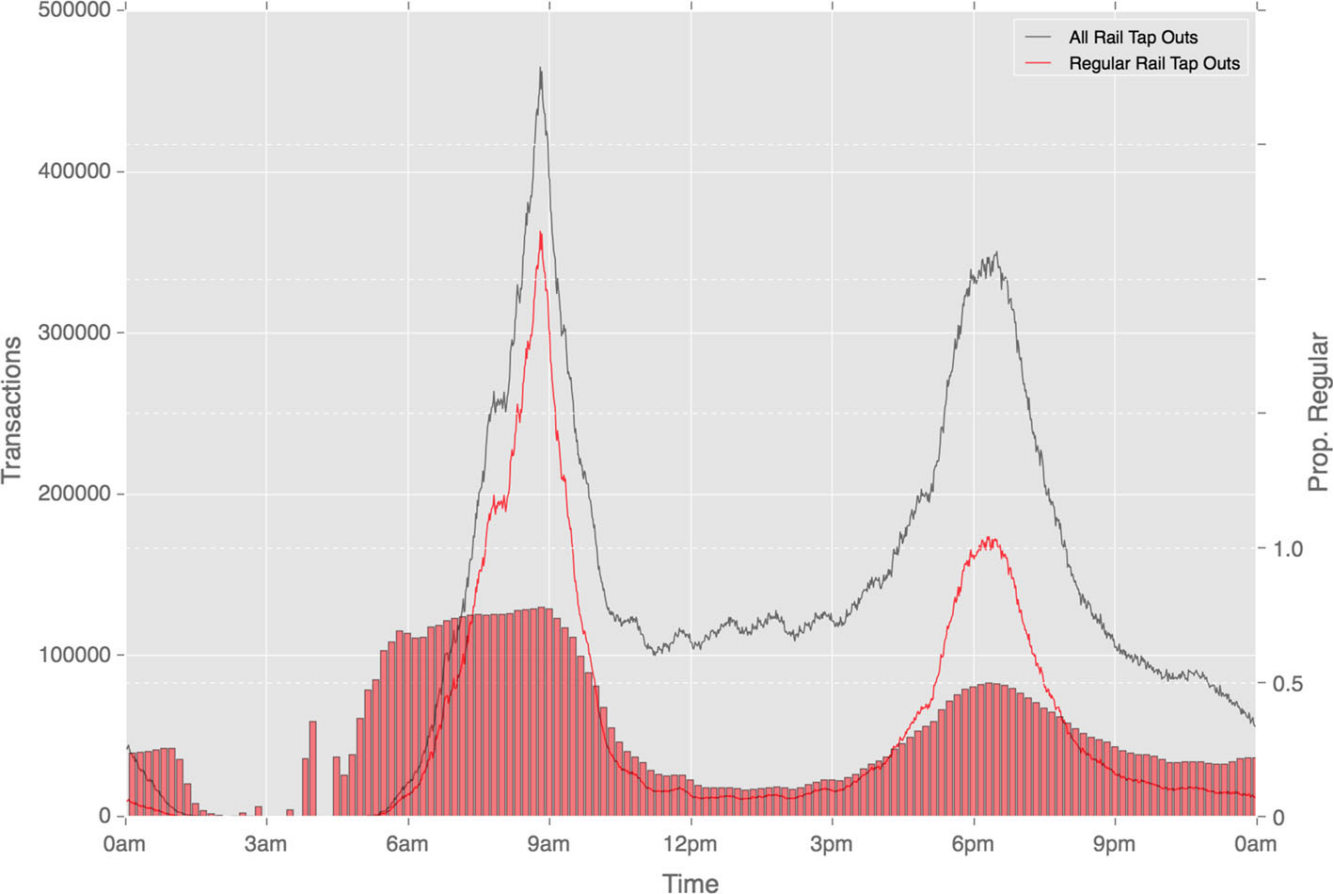
Temporal variation in regular ‘tap-out’ rail activity (absolute and relative), based on mode and origin location choice

Most of the trends in regularity observed in ‘tap-in’ behaviours on rail are repeated in ‘tap-out’ behaviours. Of the differences that are apparent, ‘tap-out’ regularity is slightly higher than ‘tap-in’ activity during the morning peak, which may be associated with travellers’ needs to reach their workplace at a specific time. As Figs. 16 and 17 show in addition to the lower number of travellers during the afternoon period, fewer of the perturbations in activity are also observed. This will be a result of the peaks in regular ‘tap-in’ activity becoming diluted as travellers reach a variety of destinations.

**Fig. 18:**
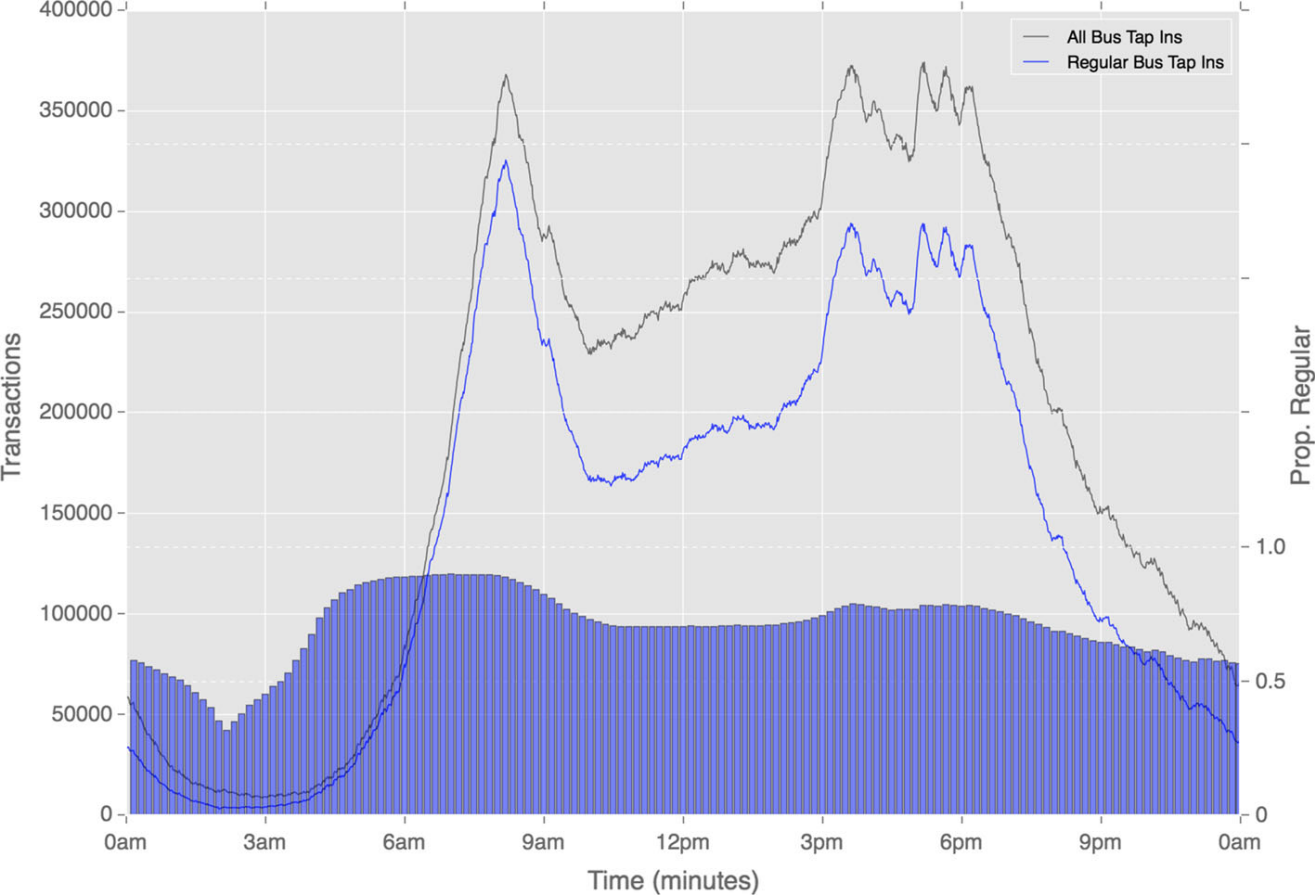
Temporal variation in regular bus activity (absolute and relative), based on mode choice alone

Greater consistency in regularity is observed on the bus network, where mode-temporal regularity is used. Unlike Underground and rail travel, regular travel on the buses remains high throughout the day, with regular trips reaching 0.73 during the Inter period and 0.57 during the Late period (contrasted with 0.37 and 0.35 respectively for relative ‘tap-in’ events). The reduction between morning and evening Peak travel is also smaller than that observed on the Underground with only an 8% reduction. As Fig. 18 shows, differences with rail activity extend further. Unlike rail travel, the strongest peak in bus activity is at around 4 pm. This suggests that regular bus use is more strongly associated with school travel, than travel by rail is. Once more, however, the half-hourly peaks that emerge during the later portion of the afternoon peak reflect the influence of work-based travel.

**Fig. 19:**
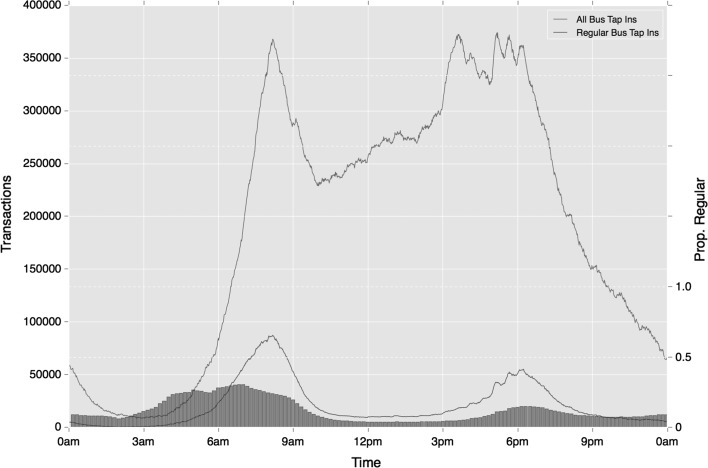
Temporal variation in regular bus activity (absolute and relative), based on mode and origin location choice

The low proportion of bus trips regularly taking place on a specific route is maintained across the course of the day, as shown in Fig. 19. While service-associated regularity increases to 0.21 during the morning peak, very few bus trips outside of peak time are classed as regular with this definition.

While some of the variation in regular travel over the course of the day may be expected, it is interesting to note the extent of variation in certain instances. While over four-fifths of travel is regular during the morning, this drops to only around 40% during the Inter period, reflecting how much ad hoc travel is conducted during this time. The degree of difference in regularity between modes is striking too. This seems to reflect how the Underground and rail are more strongly associated with regular travel during peak times only, relative to travel on the bus.

#### Day of week

A lower degree of variation is observed where trends in regularity are explored across weekdays. As can be seen from the results presented in Table 6, consistent levels in regularity are observed day-to-day across each definition of regularity. The most striking trend is the reduction in regularity from Monday to Friday, with all travel seeing a 4% decrease in the proportion of regular travel observed between these days. Between modes, once more, bus travel is observed to be more resilient to variation than underground and rail travel. Reductions in regular travel drop from 0.64 to 0.57 between Monday and Friday for ‘tap-in’ and ‘tap-out’ activity, but only fall from 0.76 to 0.73 on the buses across the same period.

**Table 6:** Proportion of trips classified as regular using each form of regularity clustering, broken down by day of the week

Classification	Monday	Tuesday	Wednesday	Thursday	Friday
All activity	0.72	0.72	0.71	0.70	0.69
Underground in	0.64	0.63	0.62	0.61	0.57
Underground in with location	0.46	0.46	0.44	0.43	0.39
Underground out	0.64	0.63	0.62	0.61	0.57
Underground out with location	0.45	0.44	0.43	0.41	0.38
Bus	0.76	0.76	0.75	0.75	0.73
Bus with service	0.11	0.11	0.11	0.11	0.10

#### Across study period

**Fig. 20:**
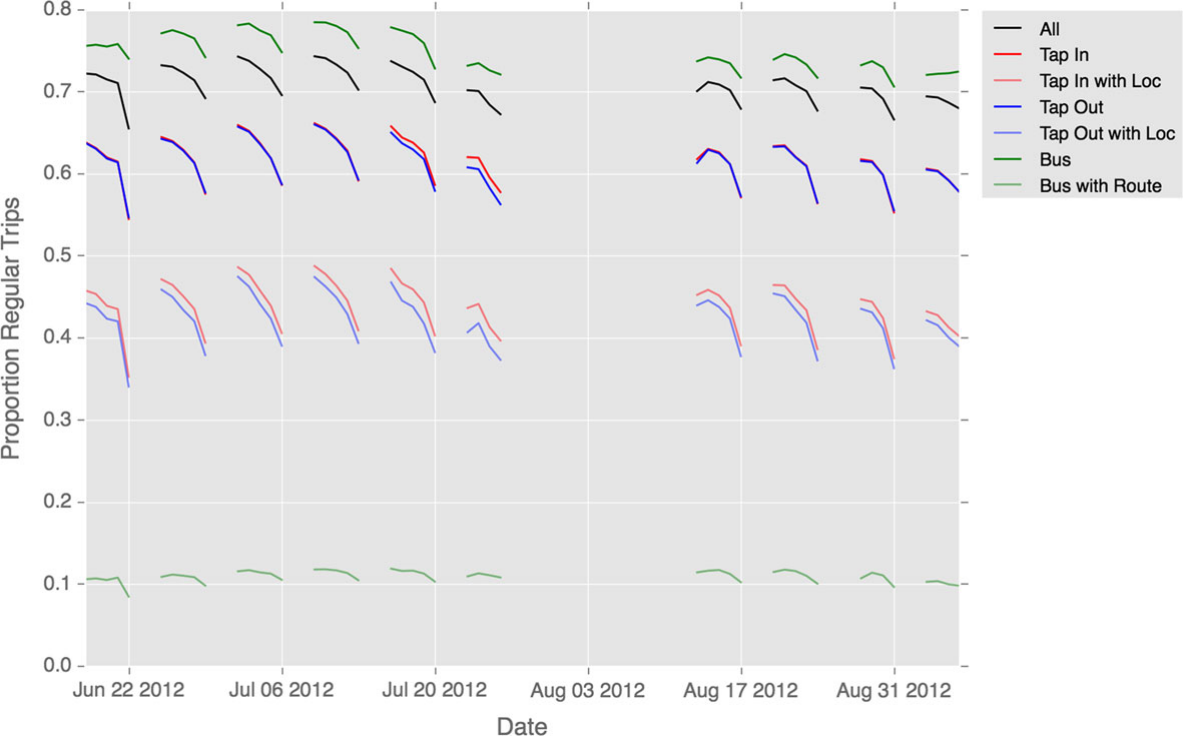
Proportion of trips classified as regular using each form of regularity clustering, shown over the course of the entire study period

The final exploration of temporal variation is carried out daily across the entire study period which begins on 16th July and ends 9th September but excludes the Olympic Games period July 27th–August 12th, 2012. The chart in Fig. 20 shows the proportion of trips considered regular by each definition, for each weekday within the study.

The results demonstrate a considerable consistency across the period. While intra-week variation is clear, as highlighted above, little inter-week variation is demonstrated. The main exception to this would appear to be the week of the 22nd July, where the proportion of regular trips is lower than usual across each day of that week. This could be explained by the beginning of the Olympics the following week, resulting in a reduction in regular travel and increase in ad hoc travel from arriving tourists. The proportion of regularity is slightly lower following the Olympics, although by only a few percentage points on each measure. This reduction is likely influenced by the impact from the school holidays beginning in late July and running through to early September.

## Discussion and conclusions

Our analysis so far has been largely descriptive but we believe that such detailed scrutiny of big data is an essential prerequisite to much more relevant and deeper understanding of travel behaviours than the current state of the art and practice reflects. We have known for a very long time that the pre-morning and morning peak are very different from the evening peak but it is very clear from our analysis here that within these broad trends and patterns, there are considerable departures from the kind of regularity that are assumed in existing models of transportation behaviour. None of this is very surprising given what we know about the increasing complexity of travel behaviour in cities with workers having ever greater flexibility over their working hours and places of work, as well as the growth of tourism in world cities which accounts for an increasing proportion of day time trips. Moreover, new patterns of significant transport to health care facilities and to schools distort the traditional morning and evening peaks, and although these patterns might show their own regularity combined with work and tourism, the whole picture can become confused. In short, different regimes of regularity can combine to generate what might appear to be quite irregular sequences of travel events. This simply points to the notion that we need other datasets associated with geodemographics and related socio-economic characteristics to give added value to this new kind of real-time streamed transit data so we can ground the patterns we have found here in less ambiguous modes of explanation. This challenge sets up a research agenda for the coming years.

We also need to move beyond examining simple clusters of travel events, toward events that are formally linked in space and time. For example, if an individual produces a cluster of events within a short time over a long period which might be interpreted as the origin events defining a journey to work, then we need to match this to destination events which might be part of a different cluster which define the concluding point of the journey to work. This kind of repetition is much harder to recognise especially if other trips are mixed with this such as visits to retail facilities and so on. Going further and matching clusters in the morning peak with the evening or at any times of the day is even more difficult to interpret as a reverse journey from work to home but it is interactions such as these which are essential to a comprehensive understanding of travel behaviour. These are all goals for the future and there are considerable possibilities of taking this analysis further using datasets of the kind we have worked with in this study. Only when we generate a much more complete understanding of regularity across all scales which involve aggregation of space/locations, times, and numbers of individuals will we be in a position to build multilevel models that begin to address the real complexity of travel behaviour. This is the message that Kitamura et al. (1997a, b) have been arguing for a number of years.

In progressing this agenda, we also need to be wary of questions involving confidentiality in individual based datasets such as the ones we have access to. Transport for London who collect and archive the data that they have provided us with have strong safeguards in place and it is impossible to identify any particular individual. But if we had a detailed classification of every kind of transportation behaviour there is, then this would be easier. We have assumed so far that every set of travel events associated with an individual in the data is unique although we know this is not the case especially once we begin to aggregate data so that the truly random occurrences associated with time when travellers tap-in and tap-out are smoothed. A bigger issue is the fact that some of the data is scrambled because there is no guarantee that each individual identifier is unique in that often individuals have more than one Oyster card and to all intents and purposes, these imply different travel behaviour. Combined with considerable loss of data due to free cards which do not require a tap-in or tap-out, as well as the conflation of season ticket holders with daily travellers, this adds to what essentially we can only interpret as noise in the data. Last but not least, we need more detailed analysis of different types of travel behaviour which are specified a priori and then used to determine whether such types exist in the data. Currently our approach is entirely deductive as is much analysis of big data – which is based on the search for pattern—rather than testing whether or not different hypotheses about how people travel can be discerned in the data. We need to extend our analysis in a more deductive manner so that we might redress the balance and introduce more explicit hypothesis testing in explaining travel behaviour.

Research around regularity should extend more widely beyond the methodology introduced in this paper. This work has focused on Oyster Card transactions alone, and therefore limited in its description of all transportation behaviours. It is likely that Oyster Card users, by virtue of the lengthier process to buying paper tickets on the London transportation system, are more regular than the remainder. One furthermore is able to gain little view into the regularity of private car drivers, cyclists, and multi-mode users. Advances in relation to these issues are becoming viable, given the movement towards universal payment through contactless payment cards (replacing both paper tickets and smart cards), and integration of transport modes through MaaS (Movement as a Service) platforms. In the shorter term, however, as has been shown elsewhere it is possible to apply these methods to other forms of spatiotemporal movement data, such as mobile phone traces. Other datasets, should they become available, may extend and support patterns derived from smart card transactions alone. While this paper has focused on the descriptive potential of regularity analysis, alternative methodologies should be explored. Advances potentially lie around alterative configurations of DBSCAN or investigations using other clustering methods for capturing individual-level regularity.

## Footnotes

1 The kernel is estimated through fitting of a Gaussian kernel to the probability distribution of the cluster length frequency dataset. Bandwidths are automatically determined using Scott’s Rule.
